# Hernia

**Published:** 1892-03

**Authors:** Wm. Steinrauf

**Affiliations:** St. Charles, Mo.


### HERNIA.

WM. STEINRAUF, M. D., ST. CHARLES, MO.

A few weeks ago I was called to see a newly-married lady of our city who was suddenly taken with severe pains in the right abdominal region, so the messenger said. After the usual examinations and explorations there was found a tumor in the right iliac region as large as a goose-egg. This had come on suddenly when trying to lift a heavy mattress, preparatory to moving into a new house. I prescribed at once, but there was no relief within the next six hours. I now put her under the influence of Chloroform and tried to reduce the rupture, but after some time of useless manipulation ceased, as the swelling would not go back. I again prescribed, but with no better success, so I sent to an allopathic surgeon of some repute. Chloroform again administered. He tried and I tried, but no result. As the friends refused to listen to an operation, I asked for a few hours more time to see what medicine would do. After again studying the case, and with much fear and trembling, I prescribed Plumbum-met. in a high potency, one dose in water every fifteen minutes. In two hours' time the tumor was gone. To say that our allopathic friend was astonished when told of the result would but faintly express it.

